# Archaeal Communities: The Microbial Phylogenomic Frontier

**DOI:** 10.3389/fgene.2021.693193

**Published:** 2022-01-26

**Authors:** Nahui Olin Medina-Chávez, Michael Travisano

**Affiliations:** ^1^ Ecology, Evolution and Behavior, University of Minnesota, St. Paul, MN, United States; ^2^ BioTechnology Institute, University of Minnesota, St. Paul, MN, United States; ^3^ Minnesota Center for the Philosophy of Science, University of Minnesota, Minneapolis, MN, United States

**Keywords:** archaea, extremophiles, microbial-communities, eukaryogenesis, rare biosphere, metagenomics, phylogenomics, archaeal phylogenetics

## Abstract

Archaea are a unique system for investigating the diversity of life. There are the most diverse group of organisms with the longest evolutionary history of life on Earth. Phylogenomic investigations reveal the complex evolutionary history of Archaea, overturning longstanding views of the history of life. They exist in the harshest environments and benign conditions, providing a system to investigate the basis for living in extreme environments. They are frequently members of microbial communities, albeit generally rare. Archaea were central in the evolution of Eukaryotes and can be used as a proxy for studying life on other planets. Future advances will depend not only upon phylogenomic studies but also on a better understanding of isolation and cultivation techniques.

## 1 Introduction

Archaea are the most genetically diverse taxa of life (Eme and Doolittle, 2015). They live in exceedingly diverse habitats, including the most environmentally extreme (Baker et al., 2020). And Archaea are foundational in the evolutionary origins of Eukaryotes (Spang et al., 2015; Zaremba-Niedzwiedzka et al., 2017). Despite their centrality in the diversity and natural history of life, Archaea were unknown for much of the early history of biological investigation. Even after the discovery of microorganisms in the mid-17th century, prokaryotic organisms were collectively known as Monera or bacteria until the mid-1970s (Woese and Fox, 1977). The discovery of two ancient lineages of prokaryotes, rather than one, overturned understanding of the history of life on Earth (Woese and Fox, 1977; Spang et al., 2017) and continues to challenge perspectives on the genetic basis of living systems (Oren, 2004). Archaeal model systems provide uniquely powerful insights into genetics and the evolution of genetic complexity (Williams et al., 2013). They provide a rich comparative genetic history since Archaea are more diverse than both bacteria and eukaryotes, demonstrating the scope of genetic and phenotypic possibilities that would otherwise be unknown in their absence.

In this review, we provide several vignettes on active research on Archaea. This review aims to shed light on vibrant and exciting research demonstrating fruitful avenues for further investigation, primarily focusing on a phylogenomic perspective on ecological and evolutionary questions. Broadly, what are the ecological and evolutionary causes, and consequences, of Archaeal diversity? We discuss the roles of Archaea in environmental communities and microbiomes, that Archaea are members of an elusive biosphere hidden in plain sight, and they are observed in unanticipated host-associated environments. We also highlight that Archaea are central to understanding the origin and complexity of eukaryotes and provide insights into the origin of life on Earth and elsewhere. The structure of each section has a historical framing to emphasize the dynamism in the field and the research opportunities. We begin with an overview of the discovery of Archaea.

## 2 The Discovery and Diversity of Archaea

More than 40 years ago, Carl Woese changed the paradigms of taxonomy and biological classification. Using ribosomal RNA (rRNA) as a molecular marker for phylogenetic reconstruction, (Woese and Fox, 1977) distinguished two separate lineages of prokaryotes from Eukaryotes. Later Woese proposed the three domains model: eukaryotes (*Eukarya*) and two prokaryotic groups (*Bacteria* and *Archaea*) (Woese et al., 1990). Of the two prokaryotic domains, *Archaea* is still the least studied; its name comes from the Greek adjective “*Archaios*,” meaning “*ancient*” or “*primitive*” (Woese et al., 1990). This clade comprises single-cell microorganisms, many of which live under extreme environmental conditions that few bacteria and eukaryotes can tolerate (Allers and Mevarech, 2005). Extreme conditions include temperature, pH range, osmotic pressure, salt concentration, and anoxic conditions (Olsen, 1994; DeLong, 1998; Gribaldo and Brochier-Armanet, 2006; Spang et al., 2017). Recent studies have revealed that Archaea are also found in mesophilic conditions, living on Earth subsurfaces, sediments, terrestrial and aquatic environments (Chaban et al., 2006; Brochier-Armanet et al., 2008; Korzhenkov et al., 2019). Archaea are essential components of hydrological systems. They play crucial roles in global biogeochemical cycling of essential redox elements, such as C, S, and N (Ramos-Vera et al., 2009; Liu et al., 2012; Offre et al., 2013), and their abundance and composition changes according to ecological spatial and temporal scales (Church et al., 2003).

Archaea are increasingly a topic of interest, especially over the last decade, because of their great physiological, metabolic morphological, and evolutionary diversity (Walsby, 1980; Stoeckenius, 1981; Eme and Doolittle, 2015). Observations of Archaeal phylogenetic richness have dramatically increased over the past several decades and especially over the last 10 years. The availability of Archaeal sequences has grown with the advent of Next Generation Sequencing (NGS) techniques and metagenomic analysis of environmental samples. In the early ’90s, only two phyla within the domain were known, Euryarchaeota and Crenarchaeota. In 2011, the TACK superphylum was proposed consisting of Crenarchaeota, Thaumarchaeota comprising ammonia-oxidizers, Aigarchaeota retrieved by metagenomics from a microbial mat in a gold mine, and Korarchaeota found in hydrothermal vents. The TACK superphylum is metabolically diverse and includes anaerobic and chemosynthetic microorganisms (Guy and Ettema, 2011). In 2013, the DPANN superphylum was Diapherotrites, Parvarchaeota, Aenigmarchaeota, Nanoarchaeota, Nanohaloarchaeota phyla, to which were later added Woesearchaeota, Micrarchaeota, and Pacearchaeota (Castelle et al., 2015). The DPANN superphylum is characterized by Archaea with small genomes, some of them symbionts (Adam et al., 2017). Members of the Asgard superphylum, another deep branching clade, were detected in estuaries (Seitz et al., 2016) and aquatic sediments (Zaremba-Niedzwiedzka et al., 2017), consisting of Thorarchaeota, Lokiarchaeota, Odinarchaeota, and Heimdallarchaeota.

**FIGURE 1:**
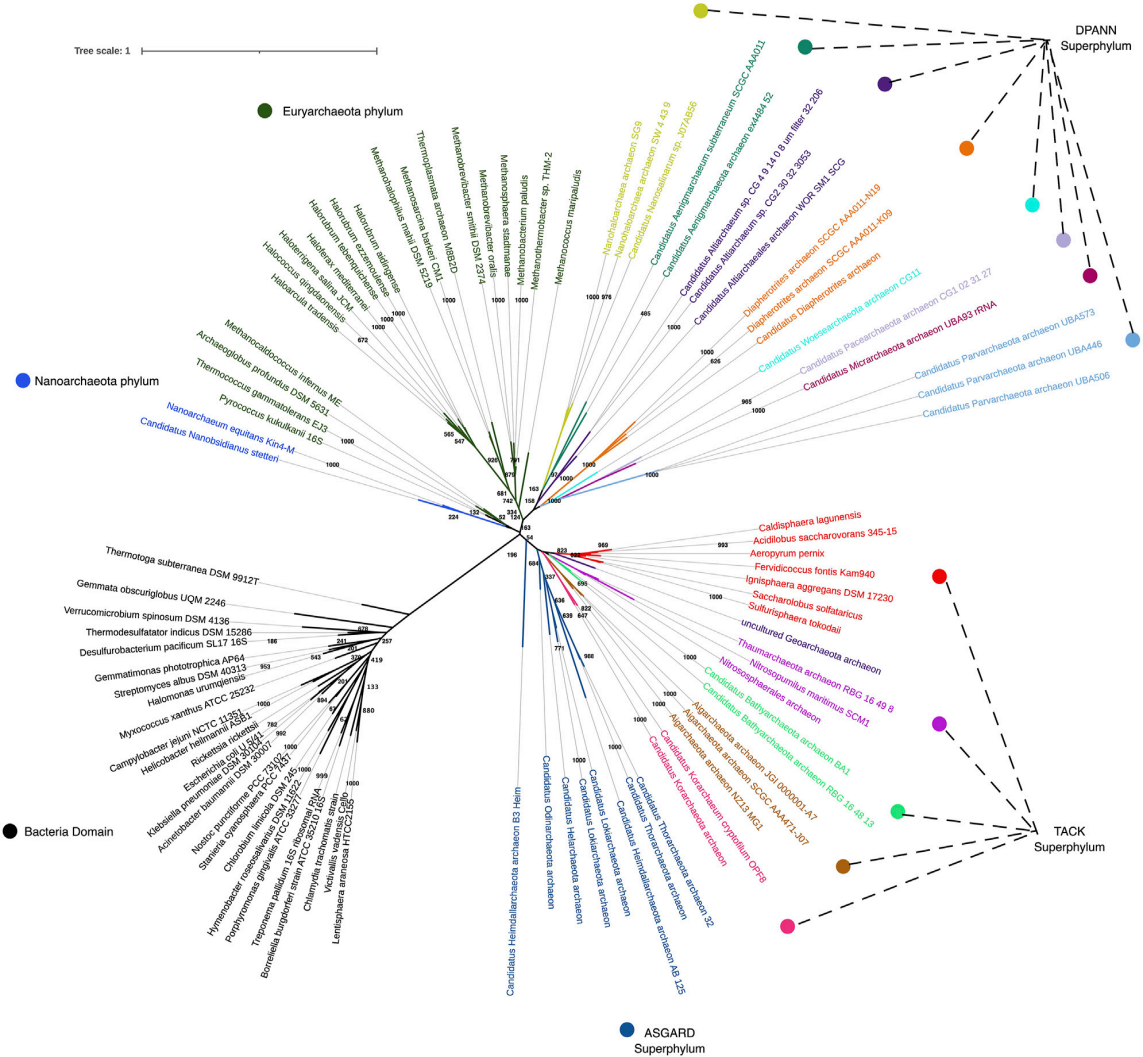
Phylogenetic tree reconstruction encompassing 16S rRNA sequences from Archaea and Bacteria domains, using the Maximum Likelihood algorithm with GTR + (G + l) evolutionary model. Reliability of inferred tree was conducted with the bootstrap test of phylogeny using 1,000 bootstraps.

One of the persistent challenges for Archaeal study has been determining their phylogenetic associations, given their genetic diversity and previously limited genetic information on some species. The Archaeal tree is rapidly expanding with new branches (see Figure 1), demonstrating that much of the Archaeal domain remains unexplored. In a recent depiction of the tree of life, encompassing the diversity of sequenced genomes, Archaea is represented by 26 phyla (Hug et al., 2016). This reconstruction summarizes all known Archaeal lineages, most of which are novel, and some include only one species of uncultivated microbe. The 30,437 genomes available from the three domains of life have been assembled through phylogenomics using a set of ribosomal protein sequences for each organism (Hug et al., 2016), unveiling the current state of genomic sampling. While most Bacteria lineages have been sampled, it appears that only 50% of the Archaeal domain has been explored (Spang et al., 2017). Even from this incomplete sampling, we can already observe that Archaea are the most metabolically diverse forms of life. And that the Asgard phyla is currently the closest relative to eukaryotes.

Phylogenomic reconstructions suggest that Archaeal lineages of the superphylum Asgard were particularly important in the organization of the eukaryotic nucleus (Spang et al., 2015; Seitz et al., 2016). The successful co-cultivation of an Asgard archaeon associated with bacteria was recently reported after long-term methane-fed bioreactor culture of deep marine sediments (Imachi et al., 2020). These non-traditional long-term cultures demonstrated that samples from untapped environments contain reservoirs of genetic and functional diversity yet to be uncovered. Due to the constant expansion of the Archaeal domain through sequencing, we have realized that traditional isolation techniques are insufficient to retrieve some microorganisms. Microbes that, for example, thrive under extreme conditions or inhabit places that are difficult to access, such as the deep ocean floors, hydrothermal vents, volcanoes, hot springs, and other sites where even sampling may represent a risk.

### 2.1 Archaea in Nature

The history of life on Earth is predominately microbial. All life was exclusively microbial for over three thousand million years from the origin of life through almost all Proterozoic (Bell et al., 2015; Tashiro et al., 2017). Even today, microbes make up most of the biodiversity of life. Since they first evolved, microbes have expanded the limits of life and have geochemically transformed the planet. Microbes are the makers and maintainers of the biosphere. They support food chains through carbon and nitrogen fixation, supply organic matter, and produce metabolites needed by multicellular organisms to grow and survive. Understanding how Archaea have shaped and continue to shape the natural world is central to investigating the organization of living systems.

In nature, microbes exist in communities as members of mixed populations, which is also the case for Archaea. Such communities frequently occur as microbial mats widely distributed in the environment and are the focus of much microbiological research. Significant efforts involve analyzing their components with different hypotheses for their construction and persistence (Baumgartner et al., 2009; Preisner et al., 2016). Microbial mats have a long history, existing for at least 75% of the Earth’s history. We know this partly because of fossil stromatolites. Stromatolites result from the lithification of organic material and sedimentary elements by microbial mats over time. The fossil record of stromatolites provides a historical narrative to evaluate existing microbial mats because their structures differ depending upon the constituent organisms (Dupraz and Visscher, 2005; Baumgartner et al., 2009).

Stromatolites are rare in the modern world but were previously as common as microbial mats are today. Mat communities from Shark Bay, Australia, have been a focus of study because it is one of the few places where stromatolites remain abundant. Using whole-shotgun 454 pyrosequencing, mat communities were observed to be dominated by bacteria, with a small number of Archaea; Crenarchaeota, Euryarchaeota, and Thaumarchaeota phyla were identified (Ruvindy et al., 2016). In 2017, a comparison of the Archaeal community was performed between two types of mats using Illumina MiSeq reads of amplicons. Smooth mats were dominated by Parvarchaeota, Euryarchaeota, Marine Benthic Group B (MBGB), and Micrarchaeota phyla, while pustular mats primarily consisted of Euryarchaeota, Parvarchaeota and Thaumarchaeota phyla (Wong et al., 2017). A comprehensive diversity-metabolic study was conducted 3 years later using Illumina NextSeq sequencing technology and Metagenome Assembly Genome (MAG). 550 MAGS were obtained; the vast majority with a phylogenetic affiliation to Bacteria, but remarkably five Archaeal genomes were assembled, including two Asgard phyla: Lokiarchaeota and Thorarchaeota, and two recently added phyla, Micrarchaeota and Woesearchaeota (Wong et al., 2018). In the previous study, Illumina HiSeq sequencing was implemented, revealing an Archaea domain proportion of 3% from the whole community, including Euryarchaeota, Thaumarchaeota, Asgardeota, and Nanoarchaeota phyla.

The studies in Shark Bay illustrate how advances in technology have led to a better understanding of Archaea. Over time, as sequencing technologies have improved, new discoveries are made possible and with greater confidence. With the aforementioned studies, better 1) sampling, 2) DNA extraction techniques, 3) NGS technologies, and 4) bioinformatics pipelines played a crucial role in the discovery of species identification and distribution. For example, more efficient and higher sampling provides better representations of microbial populations and communities (Quince et al., 2017). In addition, controlling for external contamination during sampling and nucleic acid extraction gives better estimates of sample diversity. Improved sampling also improves genome coverage and depth, leading to better genome and metagenome assembly. Higher depth contributes to robust results and the ability to detect variants with rare and *de novo* mutations. Finally, computing platforms and assembly parameters make a difference between results (Haiminen et al., 2011; Desai et al., 2013).

Studies of microbial mats elsewhere have also yielded Archaea, albeit with differing profiles. The Atacama Desert in northern Chile is well known for being the oldest existing desert on Earth (Demergasso et al., 2004; Robinson et al., 2015). It is also a habitat with a singular collection of prokaryotic diversity. Numerous surveys have identified Archaeal signatures from Euryarchaeaota, Crenarchaeota, and Thaumarchaeota phyla. These Archaea are involved in methane production, sulfur cycle, and nitrification processes, respectively, in high salinity environments (Robinson et al., 2015; Fernandez et al., 2016; Fernandez-Martinez et al., 2019). More recently, 16s rRNA gene sequences from Pacearchaeota, Woesearchaeota, and Lokiarcheota phyla and biosignatures from the TACK superphylum were detected (Saghai et al., 2017). The occurrence of these taxa is linked to oligotrophic environments such as deserts in which functional genes involved in abiotic stress are highly abundant, and genes for antibiotic resistance used in microbial competition are less abundant (Fierer et al., 2012).

The Cuatro Cienegas Basin (CCB) has a vibrant microbial fauna (Souza et al., 2006; Souza et al., 2008; Souza et al., 2018), including stromatolites and extensive microbial mats that are due, in part, to a highly skewed low phosphorous stoichiometry (Souza et al., 2018). CCB contains ∼250 permanent and seasonal pozas (ponds) and streams, all within 843.4747 km^2^ area in the Chihuahuan desert Coahuila, Mexico. Recent work at a new site in CCB (Medina-Chávez et al., 2020) identified a complex microbial mat architecture exhibiting a system of rigid gas containing hill-like domes (Archaeal Domes: AD) that rise 2–3 cm above the ground, covered by a dense, salty liquid. After three seasons (wet-dry-wet), each one represented by one metagenomic analysis on a dome (AD1, AD2, and AD3), the results show a vast diversity of members of the Archaea domain. An average of 230 Archaeal species were found in the mat (Medina-Chávez et al., 2020), covering five phyla, Euryarchaeaota, Crenarchaeota, and the unexpected Thaumarchaeota, Korarchaeota, and Nanoarchaeota phyla (Medina-Chávez et al., 2019). Total Archaeal diversity was constant through seasons, appearing as a stable Archaeal core throughout time.

Even so, more minor changes in community composition were observed. The changes in the community composition for three seasons are summarized based on the taxonomic assignment of reads. The richness between metagenomes shared 83% of the species; AD1 had 28 different species in comparison with the two others (AD2 and AD3), AD2 had one unique species that AD1 and AD3 did not have, and AD3 did not have any unique species. Phylogenetic reconstruction of species found in AD1, AD2, and AD3 was performed to display the taxonomic composition (see Supplementary Figure S1). Abundance and diversity were particularly large for Halobacteria and Methanomicrobia classes, relative to Crenarchaeota, consistent with the hypersaline microbial mat environment.

Finally, microbial mats containing Archaea are not limited to purely natural environments. For example, in Guerrero Negro, an artificial saltern located in the north of Mexico, Archaeal abundance is mainly distributed within two phylogenetic clades, Eucaryarchaeota and Crenarchaeota phyla (Robertson et al., 2009), as suggested by earlier less intensive studies (Spear et al., 2003; Orphan et al., 2008; Sabet et al., 2009; Dillon et al., 2013; Garcia-Maldonado et al., 2015). More recent sequencing validated the observation of the previously identified Archaea and greatly expanded the number of phyla. In addition, the Woesearchaeota, Nanohaloarchaeota, Diapherotrites, Aenigmarchaeota, and Lokiarchaeota phyla were also detected (Garcia-Maldonado et al., 2018).

From what we have learned about Archaea living in microbial mats and elsewhere (see below), Archaeal lineages appear widely distributed, with little evidence of geographic isolation. This raises the question: *How cosmopolitan are Archaea?* Previously, Archaea were thought to be constrained to oceans, especially on deep surfaces or hydrothermal vents, where life may have originated (DeLong, 1992; Pereira et al., 2019; Santoro et al., 2019). However, the discovery of Archaea in many locations, even in non-natural environments, demonstrates that Archaea are not limited to extreme environments and suggests that Archaea are not dispersal limited. The wide distribution of Archaea also means they may play significant roles in communities, carrying out metabolic activities that are either not available to bacteria or by doing those activities in a competitively superior manner.

The relevance of microbial mat research is not conditioned only to the identification of biogenicity. Microbial mats date back to the earliest life in the Archean eon (Nisbet and Fowler, 1999), well before many subsequent significant evolutionary changes in the history of life. The presence of Archaea in present-day mat structures provides insights into the ecological and evolutionary processes of mats in general, potentially providing a lost “fossil record” for prokaryotes. The study of Archaea found on microbial mats will likely also improve the molecular dating of evolutionary events, like divergence time estimates, speciation, and extinction rates.

### 2.2 Now You See Me, Now You Don’t

As mentioned above, Archaeal species are frequently at low relative abundances and are members of the rare biosphere. Members of the rare biosphere are often identified as genotypes with persistent sequence diversity at low relative abundance, less than <0.1%. The persistence of these rare organisms at such low frequencies raises several questions about Archaeal species and other species at very low abundances. Their low abundances may be due to a variety of ecological and evolutionary processes, including negative selection mediated by phages, banks of microbial seeds, or dormancy (Sogin et al., 2006; Jones and Lennon, 2010).

In addition, these very rare species may represent a reservoir of genetic diversity that actively responds to environmental changes (Coveley et al., 2015). Investigation of the rare biosphere is challenging. Culture-independent approaches such as metagenomics are tremendously helpful to creating prokaryotic collections. However, data for the rare biosphere is frequently discarded because of its relatively low abundance. Low abundance is especially a problem for Archaea, which are both rare and divergent from more abundant organisms. The vast majority of microorganisms have not been cultured or identified, especially in Archaea; therefore, it is not surprising new lineages with lower abundances are retrieved. Normalization of data and improved noise removal methods are essential to determine the relative abundances and identify species below 0.1% instead of defining them as sequencing artifacts. Moreover, careful manipulation, construction of references libraries, deep sequencing, robust pipelines, and technical replicates are possible solutions to the loss of taxa (Zhan and MacIsaac, 2015). The continued detection of a rare biosphere in microbial assemblages can help explain the significance of these taxa in community assembly and function despite their low abundance.

**FIGURE 2:**
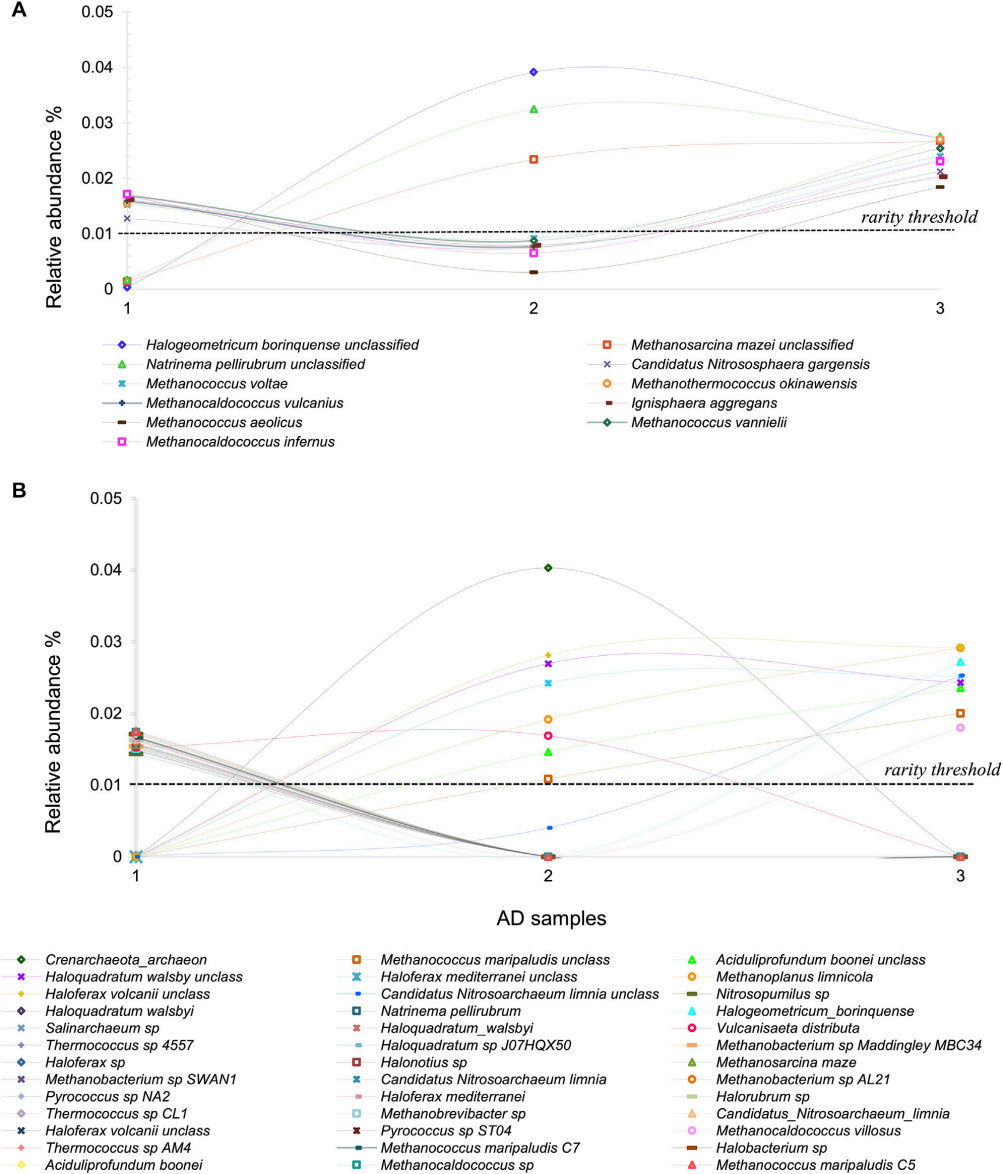
Archaeal rare biosphere from Cuatro Cienegas Basin. **(A)** Conditional rare Archaea taxa in AD in a time series, typically in low abundances but incrementing his abundance and becoming dominant in some seasons **(B)** Transiently rare Archaea taxa in AD, which tend to appear and disappear from the environment over time.

Addressing the causes and consequences of the rare biosphere requires an in-depth investigation of how rare is rare. The previous studies of Archaea at CCB provide an excellent jumping-off point on this question. By defining the “*strictly rare*” *biosphere* as the taxa with relative abundances below 0.01%, we found 50 Archaeal OTUs with very interesting demographic behavior. Within this group, 11 OTUs seem to be *conditionally rare* (Shade et al., 2014), maintaining their lower abundance in only one sampling time (the wet month) and reaching higher abundances in both dry samplings (Figure 2A) (Medina-Chávez et al., 2019). The dynamics of this portion of the rare taxa lead us to speculate that those OTUs from the Archaeal dome formation and those that are more abundant may benefit from a saltier environment under drier conditions. In both cases, it is noticeable that although Archaeal richness and abundance do not change much among samples, the rare (the one driven by environmental fluctuations) biosphere exhibits variation. A response to cold versus warm conditions has been noticed in other systems within CCB, such as in the now extinct Churince system (Rodriguez-Verdugo et al., 2012).

Additionally, 39 rare taxa were *transiently rare* since they were absent in one or two samples (Figure 2B) (Medina-Chávez et al., 2019). We suggest that this type of rarity is driven by stochastic processes such as passive dispersal of lineages temporarily recruited from the microbial seed bank or due to immigration (Lynch and Neufeld, 2015). Archaeal Domes (AD) are, so far, the most diverse microbial community found in CCB, despite the extreme conditions. Since this area is subject to water exploitation by intensive agricultural practices, desiccation and soil nutrient loss have become common in numerous ponds (Hernandez-Becerra et al., 2016). Our research group prioritizes investigating the ecology of these highly diverse Archaeal-rich microbial communities to fluctuating temperature and rainfall conditions.

It may be that the lowest-abundant taxa of the Archaeal Domes (AD) could be undergoing *dormancy* (Jones and Lennon, 2010), a mechanism that maintains cells alive but inactive and intermittently below detection thresholds*.* Archaea can enter a dormant cellular state, thereby providing an adaptive response to what would otherwise be a deleterious environmental perturbation. Dormancy has been experimentally demonstrated in the thermoacidophile archaeon *Metallosphaera prunae* that produces VapC toxins driving cellular dormancy under uranium stress (Mukherjee et al., 2017). Other stressors can induce dormancy in Archaea, including predatory organisms, specifically viruses, which are abundant in the AD metagenomes (∼28%). For example, a research group showed that rare and even inactive viruses induce dormancy in the model archaeon *Sulfolobus islandicus* (Bautista et al., 2015)*.* And dormant microorganisms can also escape virus predation (Fernandez et al., 2018). Also, low phosphorus environments, such as CCB, are enriched for dormant bacteria (Jones and Lennon, 2010).

From these studies, it is clear that much remains uncertain about Archaea and their roles in the rare biosphere. We have observed surprising dynamics in the rare biosphere. While individual Archaeal species abundances are sensitive to varying abiotic conditions, overall Archaeal abundances are not. This is consistent with the hypothesis that the rare biosphere is a reservoir for genetic diversity and that microbial communities are dependent on seasonal variation, succession, and extinction events. But so far, we have these dynamics only for the rare biosphere “community” itself. Other studies have proposed that microorganisms with low relative abundances have a significant impact in all ecosystems. Rare microbes may provide metabolic diversity when high abundance microorganisms cannot thrive under changing conditions, suggesting seasonal dynamics can be shaping community assemblage.

### 2.3 Archaea in Surprising Places

Recently, metagenomic data has greatly expanded the known distribution of Archaea, revealing the third domain is common in a wide range of environments, so much so that we can find them in the most unexpected places (Probst et al., 2013; Taffner et al., 2018). These findings have been surprising and exciting at the same time. For example, the human gut has lately been one of the most evaluated micro-environments due to microbiota-linked diseases. Several studies revealed the presence of some Archaeal members like *Methanobrevibacter smithii*, *Methanosphaera stadtmanae* as the most abundant species (Samuel et al., 2007; Dridi et al., 2009; Pausan et al., 2019) among other methanogens in low abundance, like *Methanobrevibacter arboriphilus* (Oxley et al., 2010; Khelaifia et al., 2014) and *Methanomassiliicoccus luminyensi* (Gorlas et al., 2012). Substantial Archaeal richness has also been found in animal and ruminant guts (Leahy et al., 2010; Guindo et al., 2020).

Astonishingly, human oral and tract cavities also contain Archaea populations, particularly *Methanobrevibacter oralis* and *M. smithii*, *Methanobrevibacter massiliense* (Ferrari et al., 1994; Lepp et al., 2004; Bringuier et al., 2013; Huynh et al., 2017). These species appear to be reducing CO_2_ to methane. Methanogens as *M. smithii* can also be part of the vaginal microbiome (Belay et al., 1990)*.* Moreover, human skin is also a niche for Archaea. Using PCR and specific Archaeal primers, some Euryarchaeota genera have been found, such as *Halococcus*, some unclassified Halobacteriaceae, Methanosarcina, and Methanomethylovorans; and unexpectedly, Thaumarchaota phylum is determined to be present on skin with Candidatus Nitrososphaera archaeon (Caporaso et al., 2011; Hulcr et al., 2012; Probst et al., 2013; Lurie-Weinberger and Gophna, 2015). Similarly, three phylotypes of halophilic Archaea have been detected in belly buttons (Hulcr et al., 2012). The detection of extremophile Archaea associated with humans is particularly surprising.

The rhizosphere is a complex interface (Philippot et al., 2013) with ecological dynamics impacted by the relationship with microbial communities with plant roots. Previously, the rhizosphere was thought to contain only bacteria and fungi, but recently, Archaea species have been observed. In 2018, Taffner et al. (2018), using 16s metagenomics, identified several Archaeal lineages related to bog vegetation. Methanomicrobia, Halobacteria (Euryarchaeota phylum), and Thermoprotei (Crenarchaeta phylum) classes were the most detected; *Nitrosopumilus maritimus* and *Nitrosopumilus viennensis* species (Thaumarchaeota phylum) along with unclassified taxa belonging to Thaumarchaeota and Nanoarchaeota phyla were less represented. According to functional annotations, these individuals are important constituents for CO_2_ fixation, nitrogen fixation, ammonia assimilation, besides their role as stress responders (oxidative and osmotic stress). This study also uncovered a critical involvement of Archaea in one plant-associated hormone biosynthesis, auxin, an essential plant-grow regulator for development and behavior. Other studies have shown that Archaea can substantially alter multiple aspects of a niche, allowing vegetation to grow even in harsh environments. In a study performed in the Qinghai Tibetan plateau, Thaumarchaeota lineages were retrieved from the rhizosphere, indicating ammonia oxidizers are taking a role in nitrogen fixation (Zhang et al., 2020). Nitrogen fixation takes elemental nitrogen (N_2_) and converts it into ammonia, a form usable for plants to grow.

Reciprocal evolution between Archaea and their hosts has been investigated using genomic approaches, initially for methanogenic Archaea. Newer observations have found archaeon associated with ciliates, marine sponges, and plants (Preston et al., 1996; Simon et al., 2001; Ragensbogenova et al., 2004). Some of these relationships show cross-feeding of resources and endosymbiosis, and may involve gene loss and pseudogenization events. For multicellular animals, comparative analysis of genomes revealed that archaeal endosymbionts depend on the host for the biosynthesis of essential nutrients (Lind et al., 2018), creating linked metabolisms. It has also been suggested that the dynamic between archaeal endosymbionts might be facultative since many archaeal endosymbionts can regularly be replaced by different species (Lind et al., 2018). The Archaeal common interaction with multicellular organisms dates back to early in the history of life, with divergence events within the domain, evolving and adapting to new niches. The exchange of genetic information through horizontal transfer over time explains the shared features and molecular similarities with the Eukarya domain (Staley and Caetano-Anolles, 2018).

### 2.4 Eukaryogenesis Originators

The emergence of eukaryotes is one of the most transformational evolutionary outcomes since life appeared almost 3.95 thousand million years ago (Tashiro et al., 2017). All complex multicellular life is Eukaryotic, altering the very landscape of life on Earth. Eukaryogenesis has been described as a multi-step process that occurred over millions of years of evolution (Martijn and Ettema, 2013; Dacks et al., 2016). The origin of Eukaryotes has long been a topic of speculation, with a diversity of theories and hypotheses (Koonin and Yutin, 2014; Booth and Doolittle, 2015; Lopez-Garcia and Moreira, 2020a; Lopez-Garcia and Moreira, 2020b). While endosymbiotic theories for the origin of Eukaryotes have a long history (Martin et al., 2015), the sequence of events and even the organisms involved have remained uncertain. The increasing number of novel Archaeal lineages identified by extensive genome sequencing efforts, combined with improved phylogenomic approaches, shows that the eukaryotes are highly derived descendants from Archaea and Eubacteria microbial consortia. Even so, numerous questions remain because the cellular organization of eukaryotes is far different from Archaea and bacteria, and there are no known organisms with intermediate evolutionary features.

Much of the debate on Eukaryotic origins involve the basis for Archaeal and bacterial interactions. One of the first proposed models was the eocyte hypothesis, involving predator-prey interactions between Archaea and bacteria: phagocytosis of an α-proteobacterium by an Archaea (Lake et al., 1984; Lake, 1988). Somewhat later, the hydrogen hypothesis suggested mutualistic interactions, rather than antagonism, was the basis for the first step in the origins of Eukaryotes. Martin and Müller (1998) proposed a syntrophy between a methanogenic Archaea and the α-proteobacteria as a precursor of mitochondria. Evaluation of these and other hypotheses has been challenging because of the previous lack of data on Archaea, which is now changing.

A metagenomic survey conducted on sediments near “Loki’s Castle,” a hydrothermal vent in the Arctic Mid-Ocean Ridge, revealed an Archaeal lineage (Spang et al., 2015) and the basis for the new taxa, Lokiarchaeota. The lineage belongs to a Deep-Sea Archaeal (DSAG) Marine Benthic Group B clade, a basal group within the TACK superphylum. Three DSAG bins were identified using marker genes and subsequently assembled, recovering one complete genome encoding 5,381 genes. The subsequent cultivation of a Lokiarchaeota species provides a new model for eukaryogenesis (Imachi et al., 2020), involving a two-member syntrophic co-culture composed of *Candidatus* Prometheoarchaeum syntrophicum strain MK-D1 and a species of methanogenium. Additional genomic discoveries lend support to novel ideas about the origin of eukaryotes.

There remain on-going debates about the overall topology of the tree of life. Phylogenomic analysis and physiological findings have answered some questions on the evolutionary origin of Eukaryotes, and at the same time, raised others. These analyses and their outcomes depend on implementing evolutionary models, dataset nature, and aligning methods. After Spang et al. (2015), many have contributed to the so-called 2-D (two domain) hypothesis, where Asgard superphyla is affiliated with Eukarya, suggesting Heimdallarchaeota as the closest relative Eukarya (Zaremba-Niedzwiedzka et al., 2017). Improved sampling techniques in harsh environmental conditions may result in obtaining new archaeal taxa, strengthening the tree reconstruction, and perhaps answering questions like When? How? Where? did the eukaryotes evolve (Lopez-Garcia and Moreira, 2015)?

Beyond the phylogenetic relationships between Eukarya and Asgard superphylum, both share several features, such as eukaryotic signature proteins (ESPs), including ESCRT-III homologs cytoskeletal components like actin, ubiquitin, and tubulin, which were previously thought to be exclusive to eukaryotes (Koonin, 2015). Thus, we are now far more confident that the complex endosymbiotic origin of Eukaryotes involved multiple unrelated lineages with potentially complementary ecological roles. It is also clear that the origin of eukaryotes is from a branch deep within the Archaean phylogenetic tree. And yet, there is even more uncertainty about the traits of the Eukaryotic progenitors and the topology of the Tree of Life in general.

### 2.5 Astrobiology Model System

The motivations for space exploration are complex, and yet much is captured by the hypothetical “if life could be found beyond Earth” by Joshua Lederberg (Nobel Prize 1998 for the discovery of bacterial conjugation)^1^. In the same year, the National Administration of Space and Aeronautics (NASA) was founded to look outward to space and explore other worlds. By investigating the mechanisms of the Universe, we gain a better understanding of life on Earth and the potential for life in general. NASA missions have been invaluable in discovering what is beyond our immediate reach and considering distant planetary bodies that could potentially harbor life. Perhaps surprisingly, Archaea provide a model system for understanding life elsewhere, thereby providing additional insights into life on Earth.

The detection of organic molecules in space environments such as planetary satellites, meteorites, comets, and interstellar media has provided the most significant support for the emergence and existence of life beyond our planet. This organic evidence was recorded in 1940, when CN, CH, and CH+ were recognized by optical absorption spectroscopy (McKellar, 1940). Nowadays, detecting molecular transitions in the microwave part of the electromagnetic spectrum has allowed the identification of carbonaceous compounds in distant galaxies (Ehrenfreund et al., 2006). These compounds can have prebiotic roles or contribute to the synthesis of precursor molecules and complex organics (Kwok, 2016). Identified compounds include sugars like glyceraldehyde, proteins such as formamide and acetamide, some hydrocarbons (largely methane), amines such as methylamine, and other precursors and chemical groups (Kwok, 2016). The search for life in the Universe may be limited by our definition and understanding of life on Earth (Schwieterman et al., 2018). However, the detection of similar Earth compounds in interstellar media suggests that the composition of matter may be universal, and that life on Earth can provide insight on life elsewhere Similarly, biosignatures found in space can provide a model for the early stages of life on our planet and elsewhere, taking into consideration that life and environments have changed and evolved through billions of years.

During the performance of NASA’s Curiosity rover, the mission revealed for the first time the detection of methane at different concentrations; and in 2019, methane was detected at the highest levels (https://www.nasa.gov/feature/jpl/curiosity-detects-unusually-high-methane-levels). This is an extraordinary discovery considering there are only two natural sources to the production of biogas: 1) microbial communities and 2) the interaction of some rocks and water (in this case, frozen water) (Etiope and Sherwood Lollar, 2013; Dean et al., 2018). If we hypothesize that methane gas production is due to the presence of microbial communities, the only known microorganisms capable of withstanding Martian conditions are Archaea. Moreover, the characteristic minerals on Mars are chloride and sulfate; this not only could tell how brines and salts have been part of Mars history but how extreme halophiles or poly-extremophile microorganisms can survive over time (Merino et al., 2019; von Hegner, 2020).

Archaeal methanogens provide insights on the types of microbial communities that might be found in Martian conditions, as Archaeal methanogens and halophiles are frequently found together (Nelson-Sathi et al., 2012; Watkins et al., 2014). Methanogens living in marine environments need high levels of NaCl (0.5 M) for rapid growth (Jones et al., 1987) and are usually halotolerant or halophilic (Boone et al., 1993). These methanogens use methylamines as substrates, which are produced by betaines, an accumulated osmoprotectant synthesized by halophiles (Kelley et al., 2012; Watkins et al., 2014). Both marginally and moderately halophilic methanogens such as *Methanohalobium* and *Methanohalophilus* accumulate low molecular weight solutes to equalize internal and external osmolarity, like K+, α-glutamate, β-glutamate, β-glutamine, glycine-betaine, among others (Jones et al., 1987; McGenity, 2010). For example, biogenic methane is thought to have been crucial for maintaining habitable surface temperatures before atmospheric oxygenation (Eigenbrode and Freeman, 2006; Catling et al., 2007). Thus, members of the Archaea domain are ideal models to understand osmoregulation and methanogenesis as a trace of biogenicity that possibly did occur in Mars history, offering a glimpse about the geochemical evolution of the red planet.

### 2.6 Cultivation Challenges

Moving forward, studies with Archaeal model systems will continue to take advantage of the advances in sequencing technology. All the data collected from sequences has allowed “supposing,” hypothesizing, and predicting natural phenomena occurring in this domain. It is hard to overstate how improvements in sequencing have greatly enlarged our view of Archaea. In addition, we anticipate that there will be new and more determined efforts for the isolation and growth of Archaeal species. To understand many of the phenomena in Archaea, just as in Bacteria, the physical isolation of microbes is extraordinarily helpful. While phylogenomic studies of Archaea identified patterns in their presence in environmental mats, host-associated environments, and the rare biosphere, understanding the mechanistic processes underlying those patterns is greatly aided by Archaeal isolation and culture. For example, some natural events such as adaptive evolution, species divergence, mutations, LGT/HLT (lateral-horizontal gene transfer) processes, pathogenesis, and even the large-scale production of high-value metabolites such as antibiotics would not be possible without isolation of the relevant microbes.

Isolation and culturing techniques have long been a bottleneck for studying Archaea. At present, there are several very different isolation techniques, such as streaking, plating dilution, enrichments, and media refinement for selective cultivation, most of which are derived from the history of culturing bacteria. Additional success in cultivating Archaea is likely by considering up-to-date Archaeal culturing skills and application of multiple cultivation techniques. These include co-culture, direct interspecies electron transfer (DIET), single-cell isolation, high-throughput culturing (HTC), and simulation of the natural habitat (Sun et al., 2020). A major challenge in growing Archaea is that it demands real *patience*. Rates of growth are extremely slow for some Archaeal strains, and the evidence of one single colony can take from 15 days up to 3, 6, or even 12 months. Not to mention the closest relative of eukaryotes to date, the ASGARD archaeon *Prometheoarchaeum syntrophicum* took ∼12 years until final isolation. *Prometheoarchaeum* doubling time has been estimated at around 14–25 days (Imachi et al., 2020).

Even the easiest Archaea to cultivate can be challenging. Halophilic microorganisms belonging to the Euryarchaeaota phylum are frequently cited as the easiest group to isolate since the only limiting and extreme condition is the salt concentration (NaCl). Nevertheless, despite being “the easiest” group, some challenges and technical issues still make it out of the ordinary. When culturing halophiles, it is a challenge to keep plates hydrated for 2–3 weeks or until small colonies start to appear. However, in an isolation effort done these past few years, we observed that precipitation of salts in the agar media was essential for colony growth. Even reactivation of these strains was more efficient in terms of growth when the biomass is cryopreserved along with precipitated salts.

Current isolation techniques require improvement. Although new strategies are continuously being developed, greater success can be achieved by studying Archaea physiology and metabolism through NGS functional analysis. Primary isolation is improved by mimicking the natural physicochemical conditions where Archaea thrive and by collecting the soil, water, or surface from Archaea can be retrieved. But to create an entirely whole natural niche is an impossible task. Since conditions like pH, temperature, oxygenation, and humidity can be controlled, selective and complex media design will be the key to future isolation venues. The use of transcriptomics and the modeling of metabolic networks can be proposed to facilitate media design using specific metabolites or waste products used by Archaea naturally. Furthermore, one of the achievements of Bacterial culturing is the performance of microcosm experiments and their enrichments which can also provide a greater chance to recover new and targeted archaeal species.

## 3 Discussion

It has been over 40 years since Archaea were discovered, and recognition of their importance in biology has increased tremendously. Initially, Archaea were the “other” prokaryote that was rare, exclusively living in extreme environments, and peripheral to most biology. Perceived as evolutionary oddities, they were interesting in their strangeness and that they are not bacteria. With the advent of relatively inexpensive sequencing that is increasingly powerful, a better understanding of the centrality of Archaea for life on Earth is emerging. At a minimum, the evolutionary diversity of Archaea is greater than both Bacteria and Eukaryotes combined (Staley and Caetano-Anolles, 2018). There are multiple deeply rooted lineages of Archaea that have long, apparently independent, evolutionary histories with specific environments. These lineages have the potential to provide insights into the ancient Earth before the appearance of eukaryotic, multicellular species that are likely to be inaccessible by any other means. That is over 50% of Earth’s history (Sanchez-Baracaldo et al., 2017). And because Eukaryotes are evolutionarily derived from Archaea, understanding Eukaryotes requires investigation of Archaea.

Understanding of the deep evolutionary history of Archaea, and therefore life in general, is currently in flux. The traditional view of the three domains model (Eukaryotes, Bacteria, and Archaea) hypothesized by Woese was challenged by the endosymbiotic origins of Eukaryotes involving both Bacteria and Archaea. Even so, a nuanced three domains perspective has persisted for some time, with Eukaryotes having a hybrid origin. But even this perspective is now untenable, in part because of the diversity within Archaea. The two main branches of Archaea are as deeply rooted as their separation from Bacteria, possibly suggesting a four (or more) domain model. And the relationship between Archaea and Eukaryotes is in question because Eukaryotes could be viewed more appropriately as a derived Archaeal lineage than a separate domain. These different perspectives are not likely to be resolved without additional information, which might be obtained by discovering additional diverse Archaeal lineages. However, questions about the earliest divergences across extant life could persist for some time.

While the evolutionary history of the diversity of life remains uncertain, increasing clarity is being obtained on extant Archaeal diversity. In brief, Archaea are everywhere. Frequently Archaea are rare in comparison to bacteria or eukaryotes, even when there is substantial Archaeal diversity. Archaea are often most abundant in extreme environments, even though they can also be observed in mesophilic environments. The maintenance of Archaeal diversity remains an open question, as it is for microbial species in general. Abiotic and biotic factors both contribute to changes in diversity, as for many species, yet their general rarity across most environments remains a question. *In particular, how much of their current abundance can be attributed to a long evolutionary history versus unique adaptive traits or capabilities?*

The ability of Archaea to persist in harsh environments makes them a good model system for astrobiological investigations. In the absence of life from elsewhere, Archaea provide insights into how life in challenging environments might exist. Archaea grow slowly, some very slowly. And yet, many Archaea grow under conditions that are well outside expectations for other microbes. The initial discovery of Archaea was surprising and dramatically altered our understanding of the microbial world. When considering life elsewhere and reflecting on Archaea, the pace of life may be far slower than typically observed for aerobic bacteria. And may involve environmental conditions that might be considered inhospitable, or even inimical, to other life. Recognizing the broad scope of living conditions that Archaea can inhabit can inform our investigations into life elsewhere, as well as on the early Earth. Continued identification of new Archaeal diversity is still generating surprises and is changing our perspectives, with much remaining unknown and exciting work to be done.

Reflecting over what we have already learned from Archaea, future advances are likely to enlighten biological understanding in primarily two ways, on the ecology of the existing biosphere and on evolutionary possibilities. First, our knowledge of the ecological contributions of Archaea is primarily limited to particularly harsh extreme environments, even though Archaea are found virtually everywhere. Because Archaea are so diverse, their ecological contributions are likely to be as diverse and not as narrowly constrained as initially thought. A better appreciation of the role of Archaea in ecological communities, especially those in which they are rare, provides insights into Archaeal diversity and the basis for ecological diversity itself. Archaeal distinctiveness offers a critical metric to understand ecological patterns and processes in a rapidly changing world.

The breadth of Archaeal diversity will have even greater utility in assessing evolutionary possibilities. Studying Archaea is essential for understanding Eukaryogenesis, which was a transformative event seemingly unique in the history of life and deeply dependent upon Archaea. Disentangling the basis for Eukaryogenesis will substantially enlighten our understanding of biological innovation itself. These insights include the origins of meiosis and the evolutionary benefits of recombination. More generally, the extraordinary evolutionary diversity of Archaea is a largely untapped natural experiment. Because of the large number of deep independent branches, investigations into Archaea show the potential for evolutionary possibilities that would not be otherwise available in their absence. Archaeal discoveries have repeatedly altered our understanding of the history of life, and that is likely to continue into the future.

## 4 Conclusions/Final Remarks

Archaea are a unique system for investigating the diversity of life. There are the most diverse group of organisms with possibly the longest evolutionary history of life on Earth. They exist in the harshest environments and benign conditions, providing a system to investigate the basis for living in extreme environments. Archaea were critical for the evolution of Eukaryotes and can be used as a proxy for studying life on other planets.

## Footnotes

1
https://astrobiology.nasa.gov/about/history-of-astrobiology/.

## Supplementary Material

The Supplementary Material for this article can be found online at: https://www.frontiersin.org/articles/10.3389/fgene.2021.693193/full#supplementary-material and “https://www.mg-rast.org/mgmain.html?mgpage=project&project=mgp90438
