# Supplementary figures and images for: Archaeal Communities: The Microbial Phylogenomic Frontier
Source: Front Genet. 2022 Jan 26;12:693193. doi: 10.3389/fgene.2021.693193 (PMC8826477; doi:10.3389/fgene.2021.693193)

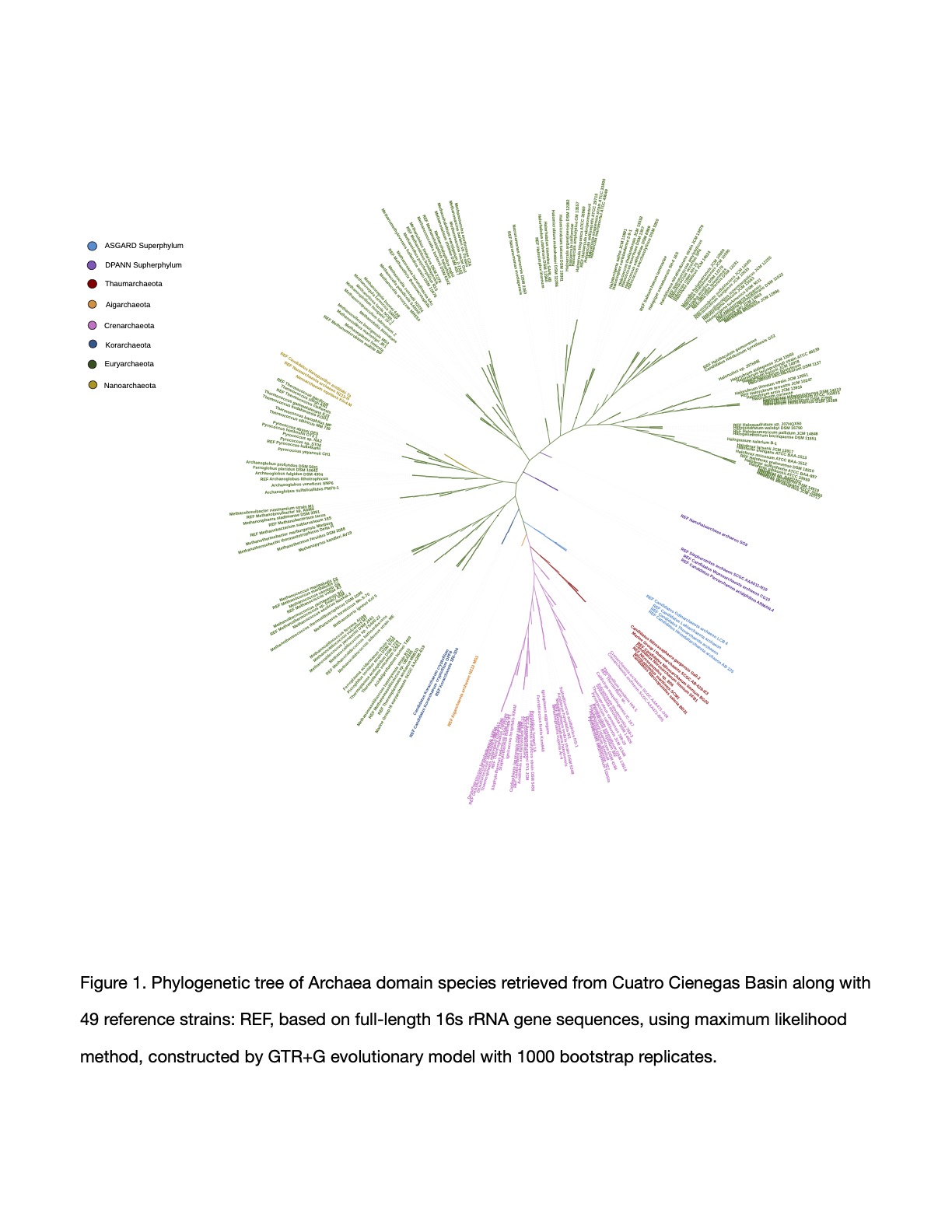

Supplement: Supplementary file 1 [file Image3.JPEG]
